# Coexistence of pulmonary tuberculosis with pulmonary sarcoidosis and skin sarcoidosis: a case report

**DOI:** 10.1186/s43162-023-00221-4

**Published:** 2023-05-15

**Authors:** Khalifa Abdulrahman Yusuf, Shadi Fayez Kanhosh, Abdulrahman Hasan Al-Madani

**Affiliations:** Department of Internal Medicine, Bahrain Defence Force Hospital-Royal Medical Services, Riffa, Kingdom of Bahrain

**Keywords:** Tuberculosis, Sarcoidosis, Corticosteroid therapy, Antimycobacterial treatment

## Abstract

**Background:**

Necrotising granulomatous diseases of the lungs exhibit a narrow range of differential diagnoses. Tuberculosis accounts for most of these cases, while sarcoidosis is an uncommon entity in this group but both possess similar clinical and radiological similarities. One must consider a diagnosis of sarcoidosis once the standard anti-mycobacterial medications fail to achieve a clinical improvement. The case described highlights the coexistence of tuberculosis and sarcoidosis which is a rare entity in the medical literature.

**Case presentation:**

A 57-year-old male presented with respiratory symptoms and was diagnosed with tuberculosis (TB) demonstrating a polymerase chain reaction (PCR) test positive showing microbial DNA in bronchial washing. The patient started standard anti-TB treatment; however, he did not respond initially. Further investigations led us to diagnose pulmonary followed by skin sarcoidosis, based on histology. After confirmation of sarcoidosis, administered corticosteroids for 6 months simultaneously along with anti-TB treatment; however, anti-TB treatment was prolonged for a total of 9 months. The patient was found clinically symptomless after the completion of treatment during subsequent follow-ups.

**Conclusion:**

The use of corticosteroids as an adjunct with standard anti-TB treatment proves beneficial effects on the recovery of patients having a coexistence of pulmonary mycobacterium tuberculosis and sarcoidosis disease conditions.

## Introduction

Tuberculosis (TB) is an infectious disease caused by Mycobacterium tuberculosis, with caseating granuloma being a hallmark. In contrast, sarcoidosis is a multisystem inflammatory disorder of unknown etiology affecting the lungs and intrathoracic lymph nodes, non-caseating epithelioid granulomas being a hallmark. Nevertheless, the similarities between conditions histologically facilitated the search for associations between both diseases. It has been considered that sarcoidosis could occur due to an immunological response toward mycobacterial antigen [1].

The main manifestations of both disease conditions are in the lungs, creating diagnostic as well as therapeutic complications; however, the coexistence of two conditions rarely occurs. Yet, some studies have reported the coexistence of pulmonary tuberculosis and sarcoidosis [2–6]. Nevertheless, therapy for both these conditions differs widely; as TB is treated with anti-tuberculosis drugs generally and only under specific indications addition of steroids, whereas sarcoidosis is treated with corticosteroids and other immunosuppressive agents [7]. Reports are also available showing the presence of mycobacterial deoxyribonucleic acid (DNA) in the tissue and bronchoalveolar lavage samples from patients with sarcoidosis [8].

It has been suggested that mycobacterial antigens could be a cause of sarcoidosis [9, 10]. Based on this, some studies have considered that tuberculosis and sarcoidosis are two different disease entities [8, 11, 12].

We report a case of the coexistence of pulmonary TB with pulmonary sarcoidosis and skin sarcoidosis. The bronchial lavage showed a polymerase chain reaction (PCR) test positive for mycobacterial DNA, while histology of bronchial and skin biopsies revealed pulmonary and skin sarcoidosis respectively. Initially patient did not respond to the anti-TB treatment alone. However, the patient showed great recovery with a dual treatment of anti-TB for 9 months and corticosteroid for 6 months.

The case is challenging at both the diagnostic as well as treatment levels since both disease conditions that occurred in the lung, share similar symptoms and histopathological features. Moreover, the decision to dual treatment showed a great improvement as the patient is clinically asymptomatic after the completion of treatment.

## Case presentation

A 57-year-old man presented with a history of shortness of breath and dry cough for 2 weeks in duration. His respiratory symptoms were of sudden onset, gradually progressive, and not associated with chest pain or palpitations. In addition, his shortness of breath was not associated with any diurnal variation. There was no history of hemoptysis, pyrexia, or weight changes. There was no history of contact with the COVID-19-positive case or tuberculosis patient. The patient took the first dose of the AstraZeneca/Covishield vaccine in February 2021; 2 weeks prior to the onset of his symptoms.

He is known to have hypertension, hyperlipidemia, diabetes mellitus type 2, and hidradenitis suppurativa diagnosed in 2006 on biological therapy for 1 year in duration.

There was no history of recent travel or exposure to industrial chemicals or gases.

There is no history of similar symptoms among family members.

On admission, the patient was normotensive with a blood pressure of 136/80 mmHg and mildly tachypneic with a respiratory rate of 20 breaths per minute and oxygen saturation of 98% on room air. Upon auscultation of his chest reduced air entry bilaterally with bibasal crepitations. The patient was not pale nor cyanosed with no signs of icterus or clubbing. All other systemic examinations were unremarkable.

On admission, routine investigations revealed a hemoglobin of 12.3 g/dL, a total count of 5440 with 59.7% neutrophils, 19.1% lymphocytes, 5.0% eosinophils, 13.8% monocytes, and a platelet count of 225,000. His erythrocyte sedimentation rate was 68 mm/h. He has normal urea and creatinine. The liver function test revealed total bilirubin of 5.5 μmol/L, alanine transaminase 18.3 IU/L, aspartate aminotransferase 16 IU/L, alkaline phosphatase 80 IU/L, total protein 72.9 g/L, albumin 37.2 g/L, and globulin 35.7 g/L (Table 1).

**Table 1:** The levels of the laboratory investigations

Laboratory investigations	November 2021	July 2021	June 2021	March 2021
Electrolytes				
Sodium (136.00–145.00 mmol/L)	136	135	138	136
Sodium (2448.00–2610.00 mg/dL)	2448	2430	2484	2448
Potassium (3.50–5.10 mmol/L)	4.56	4.61	4.69	4.25
Potassium (63.00–91.80 mg/dL)	82.08	82.98	84.42	76.50
Renal function tests				
Urea (2.76–8.07 mmol/L)	3.9	3.4	3.7	3.9
Urea (49.68–145.26 mg/dL)	70.20	61.20	66.60	70.20
Creatinine (62.0–106.0 μmol/L)	47	50	47	61
Creatinine (0.70–1.20 mg/dL)	0.53	0.57	0.53	0.64
Calcium (2.15–2.50 mmol/L)	2.33	2.61	2.7	2.37
Calcium (38.70–45.00 mg/dL)	41.94	46.98	48.60	42.66
Liver function tests				
Total protein (64.0–83.0 G/L)	82.2	92.1	85.2	72.9
Albumin (35.0–52.0 G/L)	39.4	40.4	37.3	37.2
Globulin (23.0–35.0 G/L)	42.8	51.7	47.9	35.7
Total bilirubin (0.0–24.0 μmol/L)	3	4.2	6	5.5
Total bilirubin (0.0–0.27 mg/dL)	0.03	0.05	0.07	0.06
Alkaline phosphatase (ALP) (40.0–129.0 IU/L)	134	110	81	80
Alanine aminotransferase (ALT) (0.0–41.0 IU/L)	27.2	24	21	18.3
G-glutamyl transferase (GGT) (8.0–61.0 IU/L)	100	119	116	87
Aspartate aminotransferase (AST) (0.0–40.0 IU/L)	22.4	24.2	25	16
Complete blood count				
White blood cell (4.0–11.0 × 10^9^/L)	8.41	8.53	7.02	5.44
Haemoglobin (130.0–180.0 g/L)	116	125	124	123
Platelets (150.0–450.0 × 10^9^/L)	238	322	299	225
Differential blood count				
Neutrophil (40.0–70.0%)	70.9	71.5	67.7	59.7
Lymphocytes (25.0–45.0%)	13.7	13.0	14.1	19.1
Eosinophils (1.0–9.0%)	7.4	5.4	6.4	5.0
Monocytes (5.0–10.0%)	6.3	8.7	10.1	13.8

**Fig. 1:**
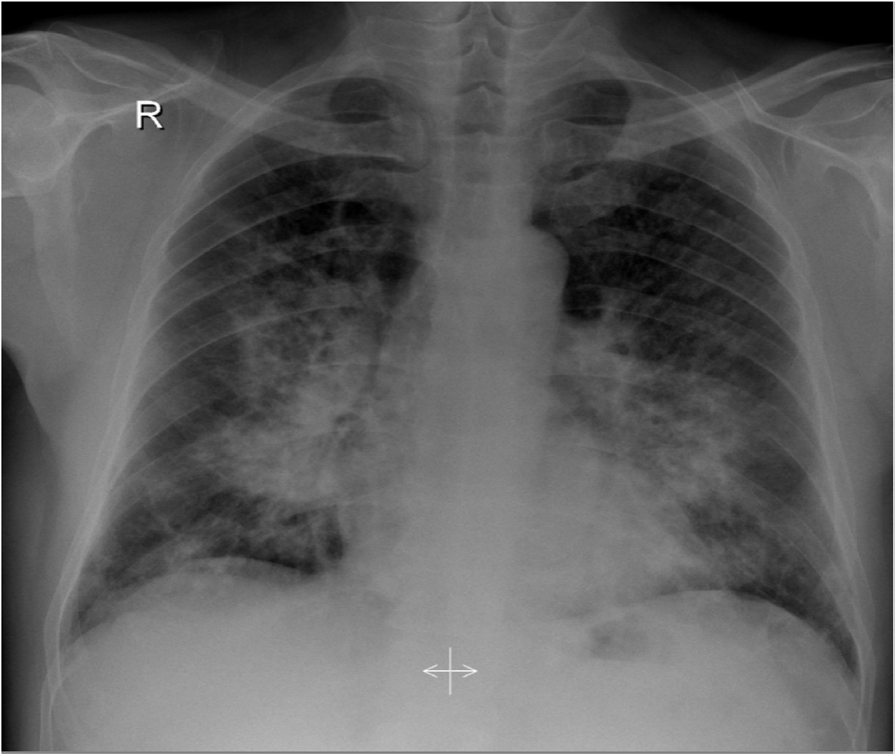
Chest X-ray showing bilateral patchy infiltration upon admission

**Fig. 2:**
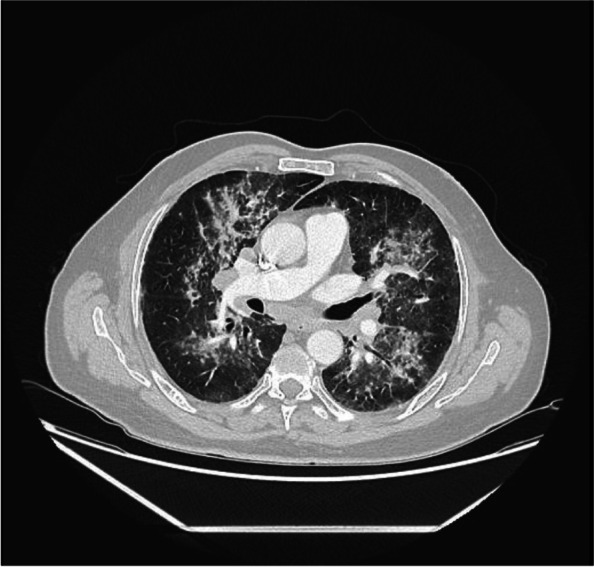
CT pulmonary angiography at presentation showing diffuse patchy consolidation with few reactive lymph nodes

Chest X-ray (Fig. 1) revealed patchy bilateral infiltration. A CT pulmonary angiography (Fig. 2) revealed bilateral diffuse patchy consolidation with no pulmonary embolus and few mediastinal reactive lymph nodes. Bronchoscopy was done, which revealed mild bilateral inflammation mainly in the right upper lobe lingula and the right middle lobe with no endobronchial lesion or mucous secretion. TB PCR Gene Expert of bronchial washing came positive for mycobacterium tuberculosis. Hence, the patient was started on Ethambutol 1200 mg OD PO, Isoniazid 300 mg OD PO, Pyrazinamide 1500 mg OD PO and Rifampicin 600 mg OD PO.

**Fig. 3:**
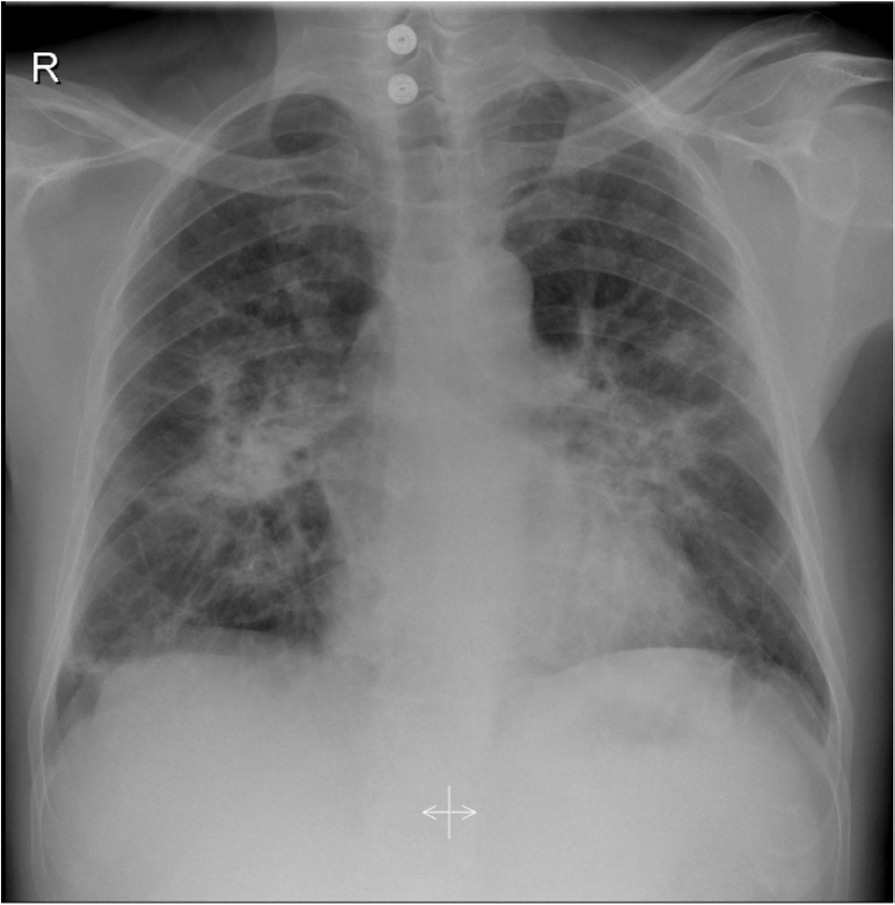
Chest X-ray showing worsening of the previously noted bilateral patchy infiltration upon recurrent admission

**Fig. 4:**
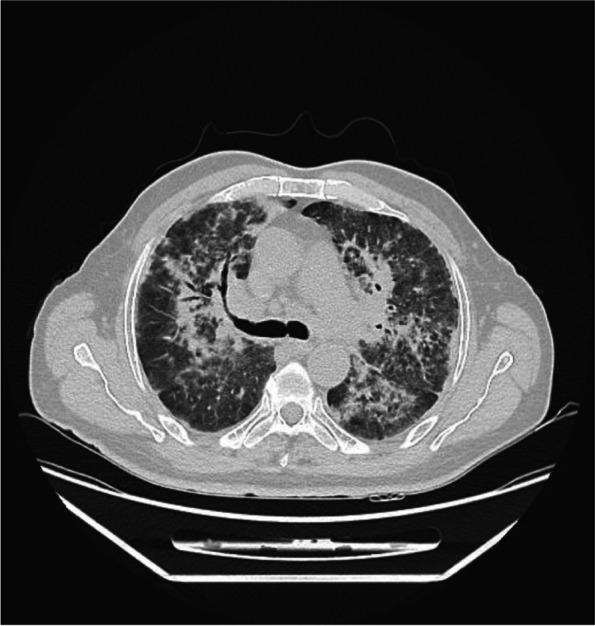
High-resolution computed tomography of the chest showing patchy bilateral areas of consolidations and ground-glass opacities with early changes of fibrosis

**Fig. 5:**
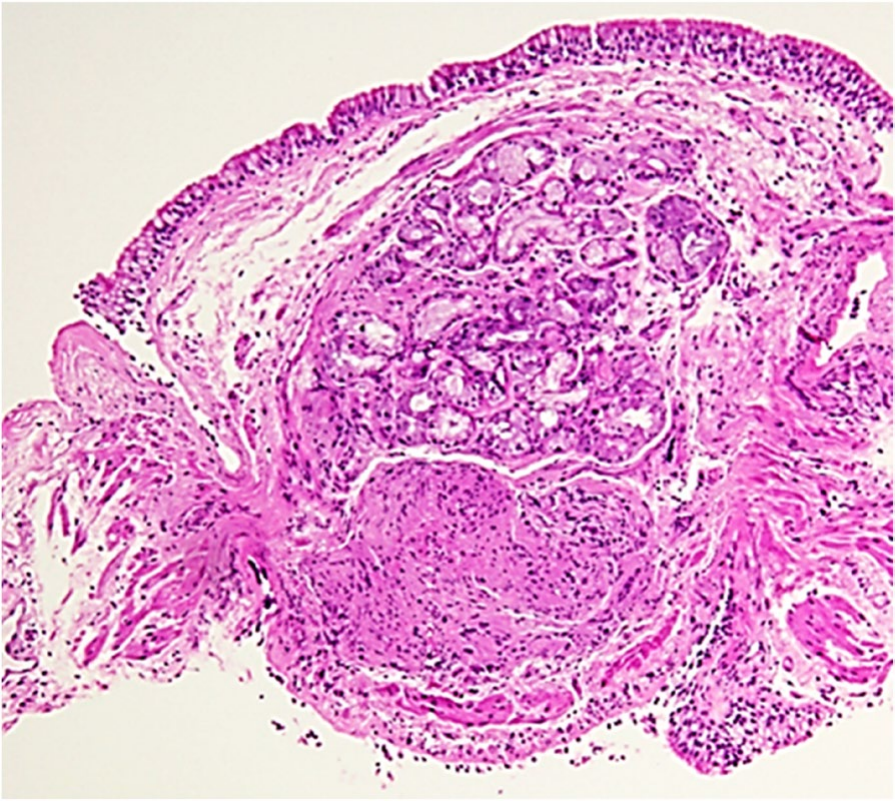
Bronchial biopsy shows a non-caseating granuloma with minimal inflammatory cell infiltrate (H&E stain)

A month later, the patient presented with similar symptoms as the previous admission with non-specific chest pain that is poorly localized and non-radiating in nature. A repeated chest X-ray (Fig. 3) was performed, which showed worsening of the patchy infiltration bilaterally. Hence, a high-resolution computed tomography of the chest (HRCT) (Fig. 4) was carried out, which revealed patchy bilateral areas of consolidation and ground-glass opacities with early changes of fibrosis and enlarged mediastinal and hilar lymph nodes. A repeated bronchoscopy was done secondary to non-resolving findings in computed tomography of the chest with a primary impression of sarcoidosis. A bronchial biopsy (Fig. 5) was done which showed a non-caseating granuloma with minimal inflammatory cell infiltrate compatible with sarcoidosis (H&E stain).

We noticed the appearance of a chronic skin lesion during the patient’s second admission after the chest symptoms. The patient was referred to dermatology for biopsied chronic skin lesions and the pathology report showed granulomatous dermatitis in keeping with sarcoidosis. Microscopic examination of the skin biopsy showed multiple granulomas in the upper dermis composed of epithelioid cells with few lymphocytes and occasional giant cells. The stain for acid-fast bacilli is negative.

After confirmation of sarcoidosis, prednisolone treatment started (30 mg OD PO) and it was tapered gradually with every subsequent monthly follow-up visit to the clinic. Treatment was given for a total of 6 months from the onset of detection of sarcoidosis.

The patient was later discharged on antimycobacterial therapy alongside corticosteroid treatment on prednisolone 30 mg OD PO. The patient was advised for repeated HRCT of the chest after two months. An extended duration of antimycobacterial treatment was recommended for a total of 9 months from initiation and tapering down the dose of prednisolone.

A pulmonary function test was carried out as an out-patient prior to the commencement of steroid therapy once the diagnosis of sarcoidosis has been established which showed an FVC of 3.36 (74% predicted), an FEV_1_ of 2.81 (78% predicted) and a TLC of 4.12 (57% predicted). Three months later, a repeated pulmonary function test was carried out which showed an FVC of 3.68 (81% predicted), an FEV_1_ of 2.92 (82% predicted), and a TLC of 3.68 (81% predicted) (Table 2).

**Table 2:** A pulmonary function test readings

	August 2021			November 2021		
	Actual	Predicted	% Predicted	Actual	Predicted	% Predicted
FVC (L)	3.36	4.51	74	3.68	4.51	81
FEV1 (L)	2.81	3.58	78	2.92	3.58	82
FEV1/FVC (%)	83.71	77.13	109	79.47	77.13	103
TLC	4.12	7.22	57	3.68	7.22	81
RV%TLC	23.88	35.80	67	37.04	35.80	103
DLCO_SB	5.69	10.16	56	7.03	10.16	69
KCO_SB	1.34	1.41	95	1.23	1.41	87

**Fig. 6:**
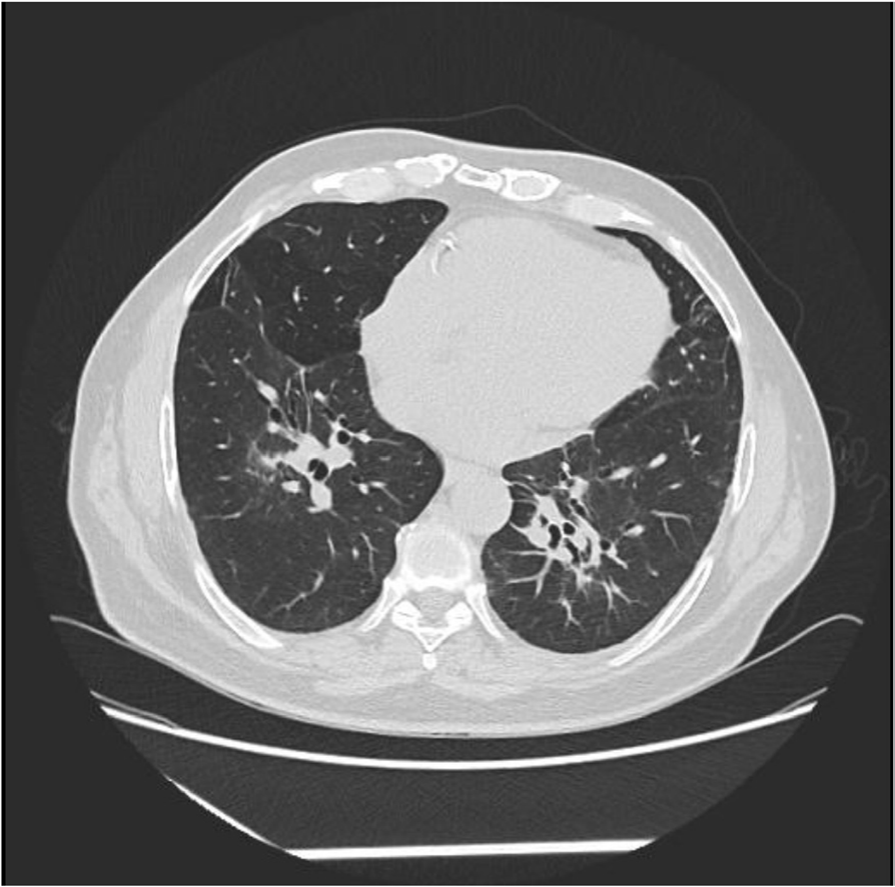
A repeated high-resolution computed tomography of the chest revealed the total subside of the previously noted diffuse pulmonary ground-glass opacities with patchy para-hilar consolidations

Repeated HRCT of the chest (Fig. 6), revealed the total subside of the previously noted diffuse pulmonary ground-glass opacities with patchy para-hilar consolidations. Currently, only residual fibrotic changes are pointed out at the interstitial level surrounding the para-hilar segmental and sub-segmental bronchi on both sides with associated traction bronchiectasis. Relative left-side pulmonary volume loss is noted due to the underlying fibrosis with obvious upward traction of the left upper bronchus and mid-portion downward kink of the left oblique fissure due to the underlying fibrotic changes.

Upon subsequent follow-ups in the clinic, the patient symptomatically improved.

## Discussion

We present the case of a patient here is that documented the coexistence of sarcoidosis and tuberculosis in the same patient. The main manifestations of both conditions occur in the lung in association with other systemic symptoms. Radiological investigations including bronchoscopy revealed positive for TB, which was supported by the presence of mycobacterial DNA in bronchial washing when performed PCR test. Hence, patient started with anti-TB treatment; however, 1 month later found no benefit to the patient, instead, HRCT showed progression in his pulmonary symptoms. Histological examination of the bronchial biopsy showed a non-caseating granuloma with minimal inflammatory cell infiltrate compatible with sarcoidosis. The absence of mycobacteria on bacteriologic examinations, the deteriorating clinical conditions of the patient while receiving anti-tuberculosis treatment, excellent response to corticosteroid therapy, and the patient was found clinically asymptotic after completion of treatment during subsequent follow-ups.

There are few reports demonstrating the coexistence of tuberculosis and sarcoidosis. Some of those reports proposed the development of tuberculosis during corticosteroid treatment of sarcoidosis [13, 14], while others described concomitant tuberculosis and sarcoidosis [15, 16]. The case presented here demonstrated tuberculosis positive, but the patient did not respond to the standard anti-TB treatment initially, further investigations resulted in sarcoidosis positive. Concomitant treatment with anti-TB and corticosteroids showed great recovery. It raised the possibility of the concomitant presence of both disease conditions.

It is important to note that the case presented here was diagnosed with hidradenitis suppurativa in 2006 and was on biological therapy (Adalimumab) for 1 year at that time, and again in 2016 the patient was on the same treatment for 4 months. Both times, before starting Adalimumab treatment, the Mantoux tuberculin skin test was performed, and it appeared negative. The patient was regular on his monthly clinical check-ups also. Considering the relatively low incidence rate of TB in Bahrain (about 15 cases per 100,000 population) [17] and the patient had no history of recent travel or history of tuberculosis in family members, we hypothesize that prolonged exposure to Adalimumab might suppress the patient’s immune system and he got a TB infection as PCR tests for mycobacterium tuberculosis DNA and bronchoscopy findings showed positive for TB in 2021, which was after 4 years of previous Adalimumab treatment. Our postulation was further supported by recent literature stating Adalimumab is a monoclonal antibody targeting the inflammatory cytokine, tumor necrosis factor-alpha (TNF-alpha) blocker that has been associated with increased risk of serious infections like tuberculosis by suppressing the host immune system [18].

After confirming the TB infection, the patient was administrated standard anti-TB treatment with wide-spectrum antibiotics.

As the symptoms occurred 2 weeks after receiving the COVID-19 vaccine, the decision to proceed with CTPA was critical to rule out thromboembolic diseases as it has been reported in the literature that the vaccine itself can cause blood clots which can manifest similarly to this patient. However, the CTPA reported negative for PE or pulmonary vein thrombosis. A connection between some COVID-19 vaccines and blood clots has been reported previously [19]. Moreover, the association between COVID-19 and TB infection has been shown as both share dysregulation of immune responses [20].

However, a month later, we found the clinical and radiological deterioration of our patient. Radiological imaging showed the ground-glass opacification, rather than alveolitis, the impression of sarcoidosis. That was further confirmed by histology on bronchial biopsy, which revealed a non-caseating granuloma, a characteristic of sarcoidosis. Ground-glass opacification has been shown to be associated with sarcoid granulomas [21].

The occurrence of eosinophilia and monocytosis can indicate the presence of a chronic inflammatory process of various aetiologies such as infectious causes, e.g., tuberculosis [22]. However, in the present case, both the eosinophil and monocyte count lie within the normal range.

Total protein, globulin, and GGT levels were raised, which is an indication of liver disease, however, total bilirubin and alkaline phosphatase were within the normal range. GGT also plays a role in breaking down drugs and toxins, as a result, levels can rise after the administration of foreign substances such as medications or alcohol, in the absence of other evidence of liver disease [23].

The histological, radiological, and clinical evidence led us to hypothesize that in the present case, TB and sarcoidosis conditions coexist. This was further supported by other reports stating the patients with TB either preceding sarcoidosis or the concomitant presence of the two conditions [3, 4, 6, 24]. Although the cause of sarcoidosis is unknown, it is proposed that a genetic predispose due to a dysregulated immune response to some provocative agents in individuals [24].

After confirmation of sarcoidosis, we started corticosteroids concurrently with anti-TB treatment which showed a great improvement in the patient’s condition. Corticosteroids are often used as an adjunct in the treatment of pulmonary and extrapulmonary tuberculosis, as combination chemotherapy potentially improves the outcomes of pulmonary TB [25]. However, the literature also reveals the negative effect on humans following corticosteroid use due to the reactivation and dissemination of TB [26, 27]. It is suggested that tuberculosis could emerge as an opportunistic infection in patients taking corticosteroid therapy to treat sarcoidosis or vaccination by bacillus Calmette-Guerin (BCG vaccine) [28–30].

In the present case, even though the patient did not show a positive response to the anti-TB treatment initially, we continued it to avoid the possibility of developing resistance to the treatment. We prolonged the duration of anti-TB medication for a total of 9 months, instead of the standard 6 months because steroids cause immunosuppression and this can increase the risk of relapse as stated previously [31].

The patient herein was referred to dermatology for biopsied chronic skin lesions and the histology revealed granulomatous dermatitis in keeping with sarcoidosis. A previous study demonstrated about 20% of sarcoidosis patients with cutaneous involvement [6]. Some types of cutaneous lesions have prognostic significance and therefore recommended to evaluate cutaneous lesions in every sarcoidosis patient for sarcoid cutaneous granulomas [32].

In the present case, our systematic follow-ups with clinical investigations led us to the appropriate diagnosis and made the right decision to introduce dual treatment simultaneously which showed great recovery, as it is evident that after completion of treatment, the patient is clinically asymptomatic.

## Conclusion

The use of corticosteroids as an adjunct with standard anti-TB treatment proves beneficial effects on the survival of patients having a coexistence of pulmonary mycobacterium tuberculosis with pulmonary sarcoidosis and skin sarcoidosis conditions. The available evidence from the present case indicates that prolonged exposure to the biological treatment-Adalimumab could predispose the host to tuberculosis infection.

## Data Availability

Not applicable.

## Abbreviations

COVID-19: Coronavirus disease 2019
CT: Computed tomography
TB PCR: Tuberculosis polymerase chain reaction
OD: Once daily
PO: Per os
HRCT: High-resolution computed tomography
H&E: Hematoxylin and eosin
FVC: Forced vital capacity
FEV1: Forced expiratory volume in 1 s
TLC: Total lung capacity
DLCO: Diffusing capacity for carbon monoxide
KCO: Carbon monoxide transfer coefficient
